# Coffin-Siris syndrome with Mayer-Rokitansky-Küster-Hauser syndrome: a case report

**DOI:** 10.1186/1752-1947-4-354

**Published:** 2010-11-08

**Authors:** Deepak Goyal, Dinesh K Yadav, Umesh Shukla, Sidharth K Sethi

**Affiliations:** 1 Department of Pediatrics and Neonatology, PGIMER & Associated Dr RML Hospital, New Delhi, India

## Abstract

**Introduction:**

We report the case of an unusual association of Coffin-Siris syndrome with Mayer-Rokitansky-Küster-Hauser syndrome. This association has never previously been reported in the medical literature.

**Case presentation:**

A nine-year-old Indian girl was referred to our hospital for growth retardation, mental retardation, lax joints, generalized hypertrichosis, and hypoplastic fifth fingernails and toenails. A thorough medical examination and evaluation revealed she had phenotypic features of Coffin-Siris syndrome, with Mayer-Rokitansky-Küster-Hauser syndrome on radiological evaluation. The karyotype of our patient was normal.

**Conclusion:**

In an unexplained case of mental retardation with facies suggestive of Coffin-Siris syndrome, association with Mayer-Rokitansky-Küster-Hauser syndrome should be considered and the patient should be evaluated for the same. Both of these syndromes may have a common pathogenesis, as yet unknown. This case report has broad implications, as similar cases in future may give insights into the pathogenesis of both these syndromes.

## Introduction

Coffin-Siris syndrome is a rare genetic disorder, also known as 'fifth digit syndrome'. In this syndrome, the most frequent findings include: mental retardation, coarse facial features, short stature, hirsutism, hypotonia, short fifth fingers, hypoplastic nails of the fifth fingers and toes, hypoplastic or absent distal phalanx of the fifth finger, and lax joints [1]. The pattern of inheritance is as yet undetermined although an autosomal recessive pattern has been suggested [1-3]. A female preponderance with a female to male ratio of 3:1 has been noted. Mayer-Rokitansky-Küster-Hauser (MRKH) syndrome, by contrast, is characterized by congenital aplasia of the uterus and the upper part (upper two-thirds) of the vagina in women showing normal development of secondary sexual characteristics and a normal 46, XX karyotype. It affects at least 1 in 4500 women [4,5]. We report the case of a 9-year-old girl with Coffin-Siris syndrome with MRKH syndrome, an association not described to date in the medical literature.

## Case presentation

A nine-year-old girl of Indian origin presented to our hospital with global developmental delay and failure to gain height. Her mother stated that she was late in sitting and standing compared to her siblings. She started sitting at one year and walking at around three years of age. Her speech was also delayed, and at nine years, she could understand only simple commands and speak few words.

She was a child of a non-consanguineous marriage, second in birth order with an uneventful birth history. Her birth weight was around 2.7 kg and her Apgar scores were normal. Her family history was unremarkable and her parents were both healthy.

**Figure 1:**
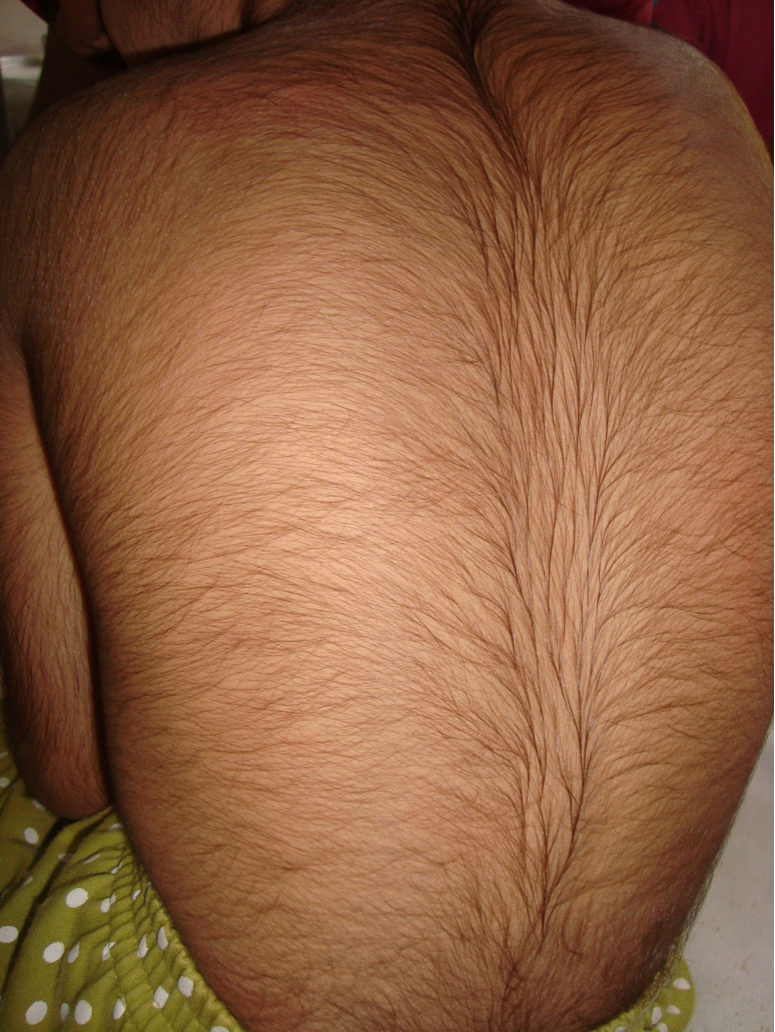
**Hirsutism of our patient**.

**Figure 2:**
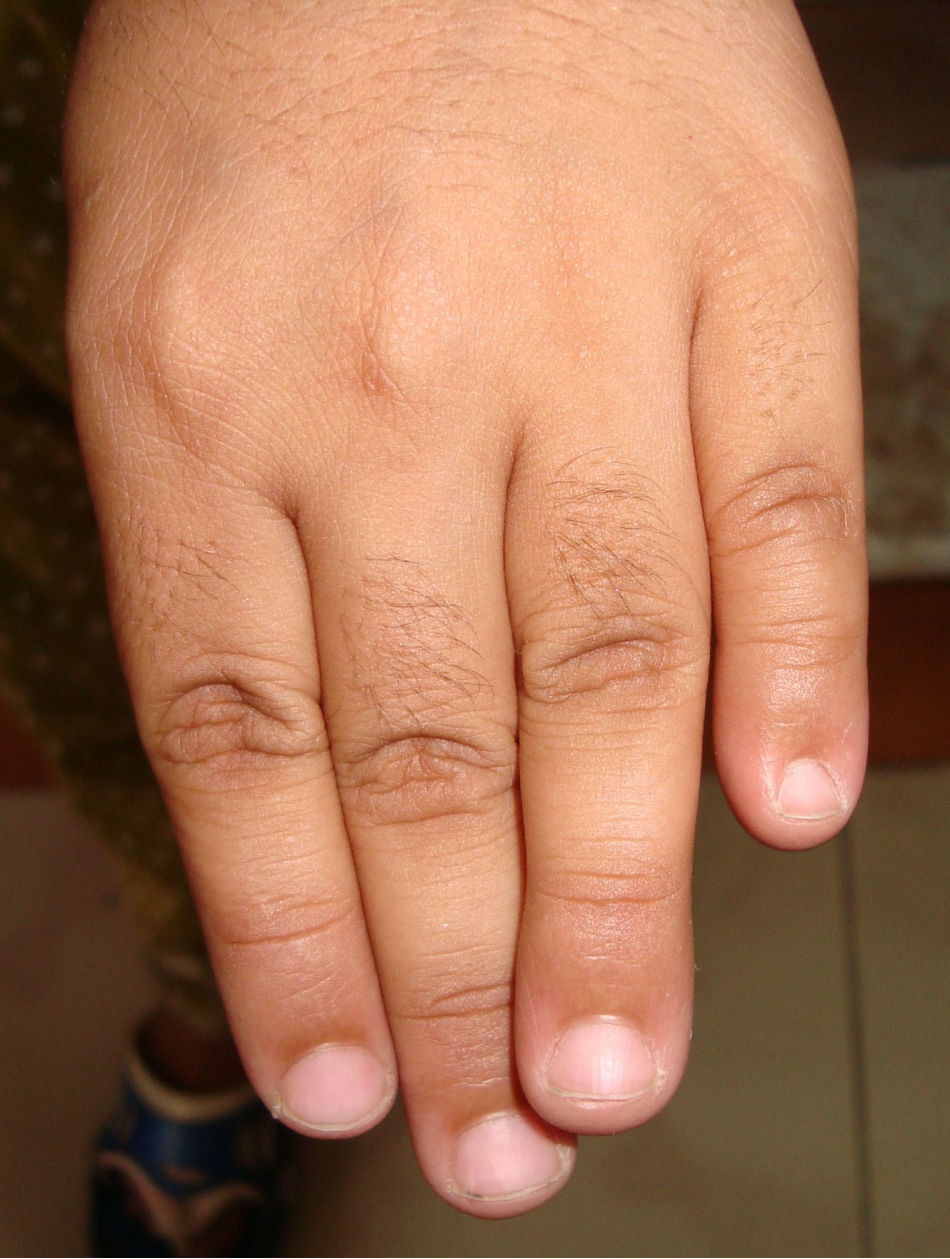
**Hypoplastic nail of the fifth finger**.

On physical examination, her height was 107 cm (below third percentile by World Health Organization standards). Her upper segment to lower segment ratio was 1:1. Her mid-parental height was 159 cm (25th percentile). She had a head circumference of 48 cm (below third percentile). She had coarse facial features, a broad nose, a thick lower lip, thick eyebrows, long eyelashes and small eyes. She had hirsutism and her scalp hair was normal (Figure 1). She had hypoplastic nails of the fifth fingers on both sides (Figure 2). Other abnormal findings include lax joints and pilonidal sinus. Neurological and other systemic examination were normal.

Her developmental examination results revealed global developmental delay (developmental quotient = 40%), more pronounced in the linguistic field. Her eye examination results revealed micro-ophthalmia and a hypermetropic disc (+6.0 dioptre in both eyes). Her ears were grossly normal. Formal audiometry tests could not be performed on our patient as she was uncooperative. Brainstem evoked response audiometry (BERA) results revealed she had normal hearing.

The results of blood chemistry tests including hemoglobin, total leukocyte count, platelets, serum creatinine, blood urea, calcium profile, liver function tests and thyroid function tests were normal. Her testosterone levels were not detectable.

The results of a radiographic examination of the hands and spine were normal. Her bone age was also appropriate for her chronological age. The results of a chest radiograph, electrocardiogram and echocardiogram were all normal. An MRI scan of her head was also normal. An MRI scan of her abdomen and pelvis revealed an absent vagina, uterus and fallopian tubes. The rest of her visceral organs were normal. Chromosomal analysis performed on the peripheral blood lymphocytes showed a normal 46, XX karyotype.

## Discussion

Coffin-Siris syndrome is a rare genetic disorder with mental retardation and absent or hypoplastic fifth fingernails and/or toenails. It is also known as 'dwarfism onychodysplasia', 'fifth digit syndrome' or 'short stature onychodysplasia'. To date around 60 cases have been reported worldwide. The first description of the syndrome was published by Coffin and Siris in 1970; they described three girls with mental retardation, absent nails of the fifth fingers and hypoplastic distal phalanges [1].

The underlying cause of this disorder is unknown. In most cases, the disorder is thought to result from genetic changes (mutations) that appear to occur randomly for unknown reasons. McGhee *et al. *[6] in 2000 published details of a patient with Coffin-Siris syndrome who had balanced reciprocal translocation between chromosome 7 and 22 (t(7;22)(q32;q11.2)). The 7q breakpoint in this case was similar to the breakpoint reported in a previous case [7] with a balanced t(1;7)(q21.3;q34). This indicates that the region 7q32>34 could be a candidate region for the gene responsible for Coffin-Siris syndrome. Kushnick and Adessa [8] in 1976 reported a case of partial trisomy 9 that phenotypically resembled Coffin-Siris syndrome. The significance of the resemblance of our patient to those with Coffin-Siris syndrome cannot be determined until more cases with both types of abnormality have been reported.

MRKH is a congenital malformation in women characterized by a failure of the Mullerian ducts to develop, resulting in a missing uterus and variable malformations of the vagina. It may be isolated (type I) but it is more frequently associated with renal, vertebral, and, to a lesser extent, auditory and cardiac defects (MRKH syndrome type II or Mullerian abnormalities, renal agenesis/ectopy and cervicothoracic somite dysplasia (MURCS) association). The etiology of MRKH syndrome is unknown, but it is believed to be due to interrupted embryological development in weeks 8 to 12 of gestation. Its inheritance pattern is unclear however in some families the condition appears to have an autosomal dominant pattern of inheritance with incomplete degree of penetrance and variable expressivity. No genes have definitely been associated with this syndrome [4,5].

The first sign of MRKH syndrome is primary amenorrhea in young women presenting with otherwise normal development of secondary sexual characteristics and normal external genitalia, with normal and functional ovaries, and karyotype 46, XX without visible chromosomal anomaly [4,5].

Other features associated with Coffin-Siris syndrome are feeding difficulties, frequent respiratory infections, scalp hypotrichosis, absent distal phalanx of fifth finger, retarded bone age and scoliosis [1,9,10]. Various eye abnormalities including myopia, astigmatism, nystagmus and strabismus have also been noted. Postnatal growth retardation and moderate developmental retardation are regular features of this syndrome [8-10]. Congenital heart disease is present in 30% of reported patients, and this includes patent ductus arteriosus, atrial and ventricular septal defects, and tetralogy of Fallot. Cleft palate and Dandy-Walker malformations have been reported in a few patients [8-10]. The degree of developmental delay and mental retardation is variable. The coarse facial features and hypertrichosis of the eyebrows may not be present at birth but may develop after early infancy. Hypotrichosis of the scalp appears to improve with age [9]. This could explain why our patient had no scalp hypotrichosis.

Since the etiology of Coffin-Siris syndrome is not yet known, the presence of an unusual combination of coarse facial features, hirsutism, growth retardation, developmental delay and hypoplastic fifth fingernails and toenails strongly suggest this syndrome. There are no laboratory tests to confirm the clinical impression and careful examination is necessary in all suspected patients.

The occurrence of both these syndromes together has never been described in the literature previously. A literature search failed to reveal any common pathogenetic origin of these two syndromes.

## Conclusions

In an unexplained case of mental retardation with facies suggestive of Coffin-Siris syndrome, an association with MRKH syndrome should be looked for, and the patient should be evaluated for the same. Both of these syndromes may have a common pathogenesis, as yet unknown.

## Competing interests

The authors declare that they have no competing interests.

## Authors' contributions

DG, SKS and US were involved in the care of our patient, relevant investigations and preparation of the manuscript. DKY supervised and helped in revision of the manuscript. All authors read and approved the final manuscript.

## Consent

Written informed consent was obtained from the parents of the patient for publication of this case report and any accompanying images. A copy of the written consent is available for review by the Editor-in-Chief of this journal.
